# MHC Class I Cross-Presentation by Dendritic Cells Counteracts Viral Immune Evasion

**DOI:** 10.3389/fimmu.2012.00348

**Published:** 2012-11-26

**Authors:** Katrin Nopora, Caroline A. Bernhard, Christine Ried, Alejandro A. Castello, Kenneth M. Murphy, Peggy Marconi, Ulrich Koszinowski, Thomas Brocker

**Affiliations:** ^1^Institute for Immunology, Ludwig-Maximilians-University MunichMunich, Germany; ^2^Department of Pathology and Immunology, Howard Hughes Medical Institute, Washington University School of MedicineSt. Louis, MO, USA; ^3^Department of Life Science and Biotechnology, University of FerraraFerrara, Italy; ^4^Max von Pettenkofer-Institut, Ludwig-Maximilians-University MunichMunich, Germany

**Keywords:** dendritic cells, cross-priming, immune evasion

## Abstract

DCs very potently activate CD8^+^ T cells specific for viral peptides bound to MHC class I molecules. However, many viruses have evolved immune evasion mechanisms, which inactivate infected DCs and might reduce priming of T cells. Then MHC class I cross-presentation of exogenous viral Ag by non-infected DCs may become crucial to assure CD8^+^ T cell responses. Although many vital functions of infected DCs are inhibited *in vitro* by many different viruses, the contributions of cross-presentation to T cell immunity when confronted with viral immune inactivation *in vivo* has not been demonstrated up to now, and remains controversial. Here we show that priming of Herpes Simplex Virus (HSV)-, but not murine cytomegalovirus (mCMV)-specific CD8^+^ T cells was severely reduced in mice with a DC-specific cross-presentation deficiency. In contrast, while CD8^+^ T cell responses to mutant HSV, which lacks crucial inhibitory genes, also depended on CD8α^+^ DCs, they were independent of cross-presentation. Therefore HSV-specific CTL-responses entirely depend on the CD8α^+^ DC subset, which present via direct or cross-presentation mechanisms depending on the immune evasion equipment of virus. Our data establish the contribution of cross-presentation to counteract viral immune evasion mechanisms in some, but not all viruses.

## Introduction

Many viruses utilize a diversity of mechanisms to evade the immune system (Tortorella et al., 2000; Yewdell and Hill, 2002). Especially herpesviruses are extremely potent immune evaders and Herpes Simplex Virus (HSV) shuts down host cell transcription, RNA splicing, and protein synthesis by expressing an arsenal of viral proteins (Hardy and Sandri-Goldin, 1994; Hill et al., 1995; Spencer et al., 1997; Song et al., 2001; Smiley, 2004). HSV-1 infects DCs with high efficiency as part of its life cycle (Coffin et al., 1998; Salio et al., 1999; Kruse et al., 2000; Mikloska et al., 2001). Consequently, infected DCs are severely compromised and fail to mature, do not upregulate expression of MHC and other surface molecules, cannot produce cytokines nor migrate properly (Salio et al., 1999; Kruse et al., 2000; Samady et al., 2003; Prechtel et al., 2005). As a consequence, HSV-infected DCs are non-functional and cannot efficiently prime naive T cells *in vitro* (Salio et al., 1999; Kruse et al., 2000).

It has been speculated that non-infected fully functional bystander DCs could cross-present exogenous viral Ag derived from infected and dying cells to secure priming of virus-specific CD8^+^ T cells (Heath and Carbone, 2001; Jirmo et al., 2009). By experimentally excluding infection of DCs, it was demonstrated that cross-presentation is principally sufficient to mediate anti-viral CD8^+^ T cell responses (Sigal et al., 1999; Norbury et al., 2001). The selective capacity of CD8α^+^ DCs to take up dead cells (Iyoda et al., 2002) and to cross-prime CD8 T cells (den Haan et al., 2000) suggests, that this DC subset may be especially important in anti-viral immunity. Indeed, several studies have found CD8α^+^ DCs to present viral Ag to CD8 T cells, when mice were infected with HSV-1, lymphocytic choriomeningitis virus, vaccinia virus, influenza virus, or respiratory syncytial virus (Allan et al., 2003; Smith et al., 2003; Belz et al., 2004a,b, 2005; Bedoui et al., 2009; Jirmo et al., 2009; Lukens et al., 2009) and ablation of CD8α^+^ DCs in Batf-3-deficient mice abrogated T cell responses to west nile virus completely (Hildner et al., 2008).

However, experiments with murine cytomegalovirus (mCMV) and viral mutants lacking immune evasion genes have not revealed substantial differences in CD8 T cell responses, leaving the question on the role of cross-presentation in the situation of viral immune evasion open (Gold et al., 2004; Munks et al., 2007). In the present study we compare immune evasion-competent HSV and mCMV with their respective mutant forms lacking inhibitory genes. We demonstrate that CD8 T cell responses to all wt and mutant viruses studied depend on CD8α^+^ DCs, which perform both, direct and cross-presentation, depending on the grade of inhibition of virus infected DCs.

## Results

**Figure 1:**
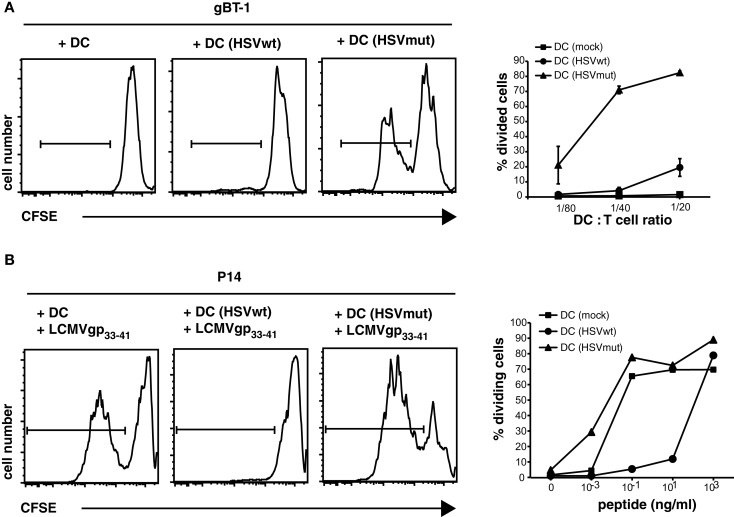
**Deletion of HSV-1 immune evasion genes rescues APC-functions of infected DCs *in vitro***. BMDC were infected with HSVwt, HSVmut, or mock-infected and utilized to stimulate CFSE-labeled TCR-transgenic HSVgB-specific CD8^+^ gBT-I T cells **(A)**. In addition DCs were loaded with the LCMVgp33-41 peptide at the indicated concentration and cultured with CFSE-labeled TCR-transgenic gp33-41-specific CD8^+^ P14 T cells **(B)**. Appropriate conditions for DC: Tcell ratio and peptide concentration were determined by titration [**(A,B)** side insets]. Main graphs show results of CFSE-profiles of gated CD8^+^ T cells for one experiment out of four with similar results in which 2500 DCs were cultured with 5 × 10^4^ CFSE-labeled T cells for 4 days and peptide concentration was 0.1 ng/ml.

To study the extent to which CD8α^+^ cross-presenting DCs can compensate immune evasion, we utilized the KOS wild type (wt) strain HSVwt, and its mutant (mut) derivative HSVmut (ΔUL41,ICP4,22,27), which lacks four viral genes responsible for different viral “immune evasion”-strategies (Krisky et al., 1998; Lauterbach et al., 2004). *Vhs*, the *virion host shutoff protein* encoded by the gene UL41 causes destabilization and degradation of infected host cell mRNAs and is among other effects responsible for down regulation of MHC I synthesis and expression (Hill et al., 1994; Tigges et al., 1996; Hinkley et al., 2000; Koppers-Lalic et al., 2001). ICP4 reduces the stability of host cell mRNA (Mogensen et al., 2004), ICP22 modifies the host RNA polymerase II (Rice et al., 1995), while ICP27 inhibits mRNA biogenesis leading to shutoff of host protein synthesis (Hardwicke and Sandri-Goldin, 1994; Hardy and Sandri-Goldin, 1994). Deletion of these viral genes rescued maturation and immune functions of infected human monocyte-derived DCs *in vitro* (Samady et al., 2003). To confirm the effect of these viral genes on capacities of murine DCs to prime CD8^+^ T cells *in vitro*, we infected bone marrow derived DCs (BMDCs) with wt HSVwt or HSVmut and utilized them as APC for naïve CD8 T cells. The priming of naïve HSV-glycoproteinB (gB)-specific CD8^+^ T cells by HSVwt-infected BMDCs was extremely inefficient and only at the highest DC: T cell-ratio very few divided cells could be detected (Figure 1A). In contrast, when BMDCs were infected with HSVmut, they were highly efficient to prime T cells to divide (Figure 1A). To exclude the possibility that these results were caused by different amounts of viral gB-antigen being expressed by cells infected with the different variants of HSV, we loaded infected DCs with titrated amounts of an HSV-irrelevant LCMVgp_33-41_ peptide and tested their capacity to prime specific CD8^+^ P14 T cells (Figure 1B). HSVmut-infected DCs were as efficient in priming naïve P14 T cells as non-infected mature DCs (Figure 1B). In contrast, HSVwt-infected DCs needed 10^4^ times higher peptide concentrations to induce comparable CD8^+^ T cell priming (Figure 1B). These results show that deletion of these viral genes can rescue the capacity of infected DCs to prime CD8^+^ T cells *in vitro*.

**Figure 2:**
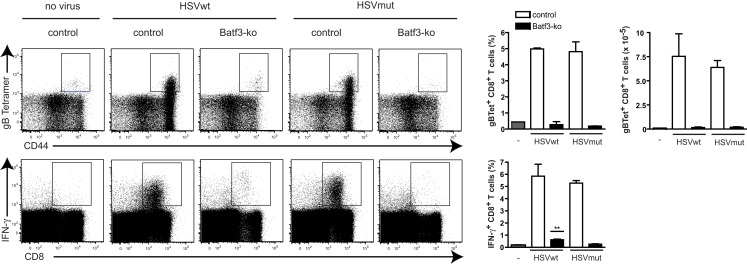
**Batf3-ko mice cannot mount HSV-specific CD8 T cell responses**. Batf3-ko and C57BL/6-mice were infected i.v. with 4 × 10^6^ infectious particles of HSV. Mice were sacrificed 5 days later and spleens were analyzed for presence of HSVgB-specific CD8^+^ T cells with H-2 K^b^/gB_498-505_-tetramers (top panel) or by *ex vivo* restimulation with gB_498-505_ peptide and subsequent intracellular FACS-analysis for IFNγ-production (lower panel). Dot plots are gated on CD8^+^ T cells (upper panel) or lymphocytes (lower panel) first (not shown). Data shown as bar graphs are mean ± SEM from *n* = 3 mice per group. (**P = 0.0018 as compared to unstimulated control). Open bars represent C57BL/6-wild type mice and filled bars represent Batf3-ko mice. This experiment has been repeated twice with similar outcome.

Next we tested, if these different effects of wt- and mut-viruses on DCs were of relevance for *in vivo*-priming of virus-specific CTL and investigated the role of CD8α^+^ DCs. Comparative studies between wt virus and mutant variants are generally difficult to interpret due to different and partially uncharacterized viral properties. Therefore we compared in the following studies only CD8^+^ T cell responses to same virus, either wt or mut, but in different mouse strains. To study the role of CD8α^+^ DCs, we infected *Batf3^−/−^* mice, which lack the transcription factor Batf3 leading to defective development of CD8α^+^ and CD103^+^CD11b*^−^* DCs, the two major cross-presenting DC-subpopulations (Hildner et al., 2008; Edelson et al., 2010). As HSVmut does not interfere efficiently with immune functions of infected DCs (Figures 1A,B), we speculated that CD8^+^ T cell responses against this viral mutant should be more independent of cross-presentation than those induced by HSVwt. While C57BL7/6 mice mounted strong HSVgB-specific CD8^+^ T cell responses as measured with the respective MHC-tetramers, we could not detect significant gB-specific CD8^+^ T cells in HSVwt-infected Batf3*^−/−^*-mice at all (Figure 2, upper panel). Surprisingly, also HSVmut, despite of being devoid of major inhibitory genes, could not elicit gB-specific T cell responses in Batf3*^−/−^*-mice, while it did so very efficiently in wt mice (Figure 2, upper panel). When the spleens of the same animals were monitored for IFN-γ-producing CD8^+^ T cells, only very low frequencies of IFN- γ^+^ CD8^+^ T cells were detectable in HSVwt-infected Batf3*^−/−^*-mice (Figure 2, lower panel). IFN-γ-producing CD8^+^ gB-specific CD8^+^ T cells were not present in HSVmut-infected mice at all (Figure 2, lower panel). This data indicates that the DC-subpopulations lacking in Batf3*^−/−^*-mice are central for inducing CD8^+^ T cell responses upon infection with HSVwt and HSVmut.

**Figure 3:**
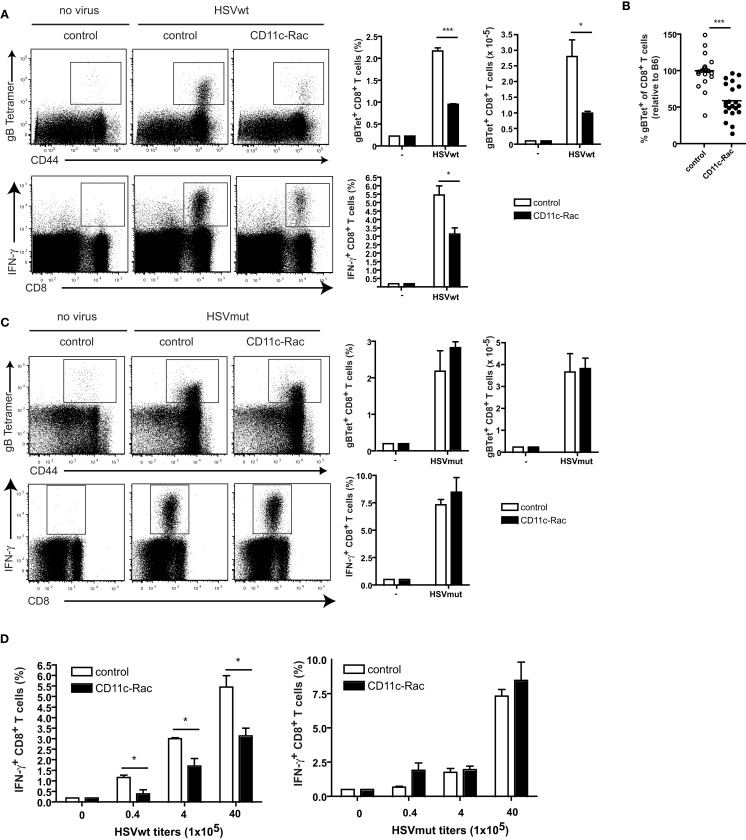
**Cross-presentation deficient CD11c-Rac mice mount reduced CD8 T cell responses to HSVwt, but normal responses to immune evasion-deficient HSVmut**. Mice were immunized i.v. with 4 × 10^6^ infectious particles **(A,B,C)** or graded amounts **(D)** of HSVwt or HSVmut and 5 days later CD8^+^ T cells from spleens were analyzed as described in Figure 2. Dot plots are gated on CD8^+^ T cells [**(A,C)** upper panel] or all lymphocytes [**(A,C)** lower panel] (not shown). Data from one out of four experiments with similar results (*n* = 3 mice per group) are shown as mean ± SEM in bar graphs (a, c; side insets), *t*-test analysis, a, upper panel: ***P < 0.0001; *P = 0.0284; a, lower panel *P = 0.0243. **(B)** Pooled data from seven experiments (*n* = 3 mice per group, total *n* = 21) are displayed as percent of control (C57BL/6). The mean of each control group was set to 100% and the data from the CD11c-Rac-groups were calculated relative to the respective control group (***P = 0.0001). **(D)** Mice were immunized with the indicated amounts of HSVwt or HSVmut. Analyses were performed as described in **(A)**. HSVwt 0.4 × 10^5^, *P = 0.0221; 4 × 10^5^, *P = 0.0223; 40 × 10^5^, *P = 0.0243; One out of two experiments with similar outcome is shown (*n* = 3 mice per group).

In order to discriminate between the contributions of direct vs. cross-presentation we next infected CD11c-Rac mice with HSV viruses. In DCs of these mice, the dominant negative N17Rac-transgene causes inhibition of uptake of exogenous soluble, cellular, or apoptotic antigen leading to strong reduction of cross-presentation (Kerksiek et al., 2005). As a consequence, CD11c-Rac mice show deficient peripheral tolerance to self proteins (Luckashenak et al., 2008), absent CD8 T cell responses to extracellular bacteria, to cell associated proteins, to apoptotic, or soluble protein antigens (Kerksiek et al., 2005; Neuenhahn et al., 2006; Luckashenak et al., 2008). Upon infection with HSVwt-virus we could detect approximately half the frequency and numbers of HSVgB-specific CD8^+^ T cells in spleens of cross-presentation-defective CD11c-Rac mice, as compared to non-transgenic littermates (Figure 3A). Both, detection of specific T cells by MHCI-tetramers (Figure 3A, upper panel), as well as analysis of IFN-γ-producing CD8^+^ T cells upon restimulation with viral HSVgB-peptide *in vitro* (Figure 3A, lower panel) revealed strongly reduced frequencies and total numbers of HSV-specific CD8^+^ T cells in CD11c-Rac mice. These differences were highly significant, also when data from several independent experiments were pooled (Figure 3B). In marked contrast, infection with HSVmut elicited comparable frequencies and total amounts of HSVgB-specific IFN-γ-producing CD8^+^ T cells in CD11c-Rac- and control mice (Figure 3C). These data indicate, that lack of inhibitory genes in HSVmut allows normal priming of HSVgB-specific CD8^+^ T cells independently of cross-priming in CD11c-Rac mice. It has been speculated that CD8^+^ DCs preferentially capture dying cells (Iyoda et al., 2002) and efficiently cross-present relatively high-dose tissue-associated antigens (Kurts et al., 1998) as probably occur during viral infections. To test if dependency on cross-presentation was observed also upon inoculation with low doses of virus, we infected mice with graded amounts of HSVwt or HSVmut (Figure 3D). Our data indicate that independently of the amount of virus utilized for infection, the priming of approximately half of all HSV-specific CD8^+^ T cells depends on cross-presentation, as the responses to infection with HSVwt were reduced approximately 50% in CD11c-Rac mice (Figure 3D). However, when viral immune evasion genes were deleted in HSVmut, the amount of primed HSVgB-specific CD8^+^ T cells in CD11c-Rac mice was indistinguishable from those observed in wt mice (Figure 3D).

**Figure 4:**
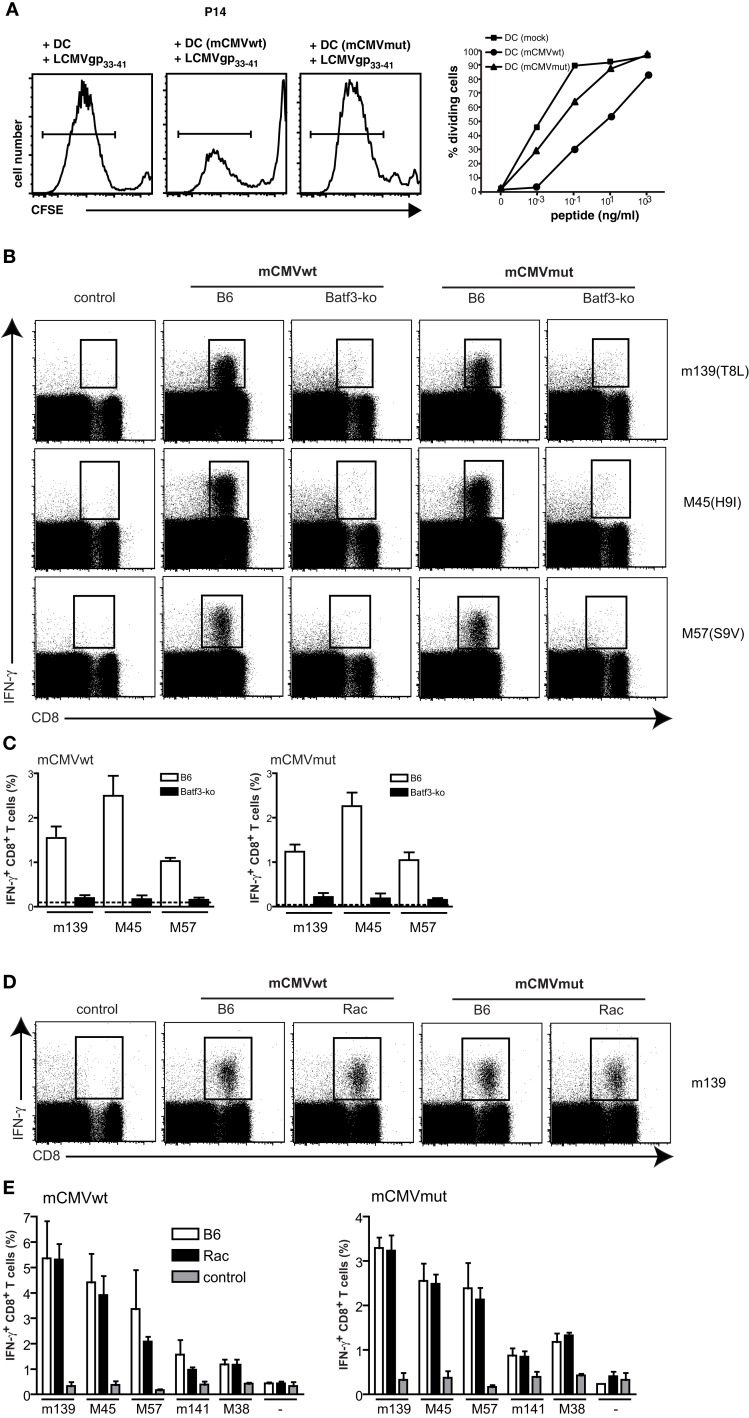
**mCMV responses do not depend on cross-presentation**. BMDC were infected with mCMVwt, mCMVmut, or mock-infected. DCs were utilized to either stimulate 5 × 10^4^ CFSE-labeled TCR-transgenic gp33-41-specific CD8^+^ P14 T cells as described in Figure 1B **(A)**. CFSE-profiles of gated CD8^+^ T cells are shown for the peptide concentration 0.1 ng/ml. Graphs show results of gated divided T cells of one experiment out of two with similar results. **(B)** Batf3-ko and C57BL/6-mice were infected i.v. with 4 × 10^6^ infectious particles of mCMVwt or mCMVmut. Mice were sacrificed 7 days later and spleens were analyzed for presence of mCMV-specific CD8^+^ T cells by *ex vivo* restimulation with the indicated mCMV-peptides and subsequent intracellular FACS-analysis for IFNγ-production. Dot plots are gated on lymphocytes first (not shown). **(C)** Data from **(B)** shown as bar graphs are mean ± SEM from *n* = 3 mice per group. Open bars represent C57BL/6-wild type (wt) mice and filled bars represent Batf3-ko mice. This experiment has been repeated twice with similar outcome. **(D)** CD11c-Rac- or C57BL/6-mice were infected i.v. with 4 × 10^6^ infectious particles of mCMVwt or mCMVmut. Mice were sacrificed 5 days later and spleens were analyzed for presence of mCMV-specific CD8^+^ T cells by *ex vivo* restimulation with the indicated mCMV-peptides and subsequent intracellular FACS-analysis for IFNγ-production. Dot plots are gated on lymphocytes first (not shown). **(E)** Data from **(D)** shown as bar graphs are mean ± SEM from *n* = 3 mice per group. Open bars represent C57BL/6-wt mice, black bars represent Batf3-ko mice, gray bars are non-immunized controls. This experiment has been repeated twice with similar outcome.

We next tested if murine CMV (mCMVwt) and its triple mutant mCMVmut, which is devoid of several MHC class I inhibitory genes would be similarly dependent on cross-presenting CD8α^+^ DCs. The mutant form of mCMVwt (C3X), mCMVmut (Δm04Δm06Δm152; Wagner et al., 2002), lacks three known “immunoevasins” that act on the MHC class I presentation pathway. While m04 binds to MHC class I (Kleijnen et al., 1997) and prevents activation of T cells (Kavanagh et al., 2001), m06 retargets MHC class I to lysosomal degradation (Reusch et al., 1999) and m152 retains MHC I/peptide complexes in the ER-cis-Golgi compartment (Ziegler et al., 1997) leading to an overall reduction of MHC class I surface expression. Accordingly, when mCMVwt- or mCMVmut-infected gp33-41 peptide-pulsed BMDC were cocultured with gp33-41 peptide-specific P14 T cells, the mCMVmut-infected DCs induced more efficient T cell responses as compared to DCs infected with mCMVwt. However, in comparison to the HSV study (Figures 1A,B), the overall inhibitory effect of mCMVwt virus was weaker as compared to HSV (Figure 4A).

In analogy to the experiments with HSV described above, we next infected wt and Batf3-ko mice with the two viruses and measured IFN-γ producing virus-specific CD8 T cells. CTL-responses against both, mCMVwt and mCMVmut, entirely depended on the presence of CD8α^+^ DCs, as we could not detect mCMV-specific CD8 T cells in Batf-3 ko mice immunized with either virus (Figures 4B,C). To further analyze if mCMV-specific CD8 T cells would be primed via direct or cross-presentation by CD8α^+^ DCs, we infected CD11c-Rac mice and compared the responses to those elicited in non-transgenic littermates (Figure 4D). However, and in contrast to the results obtained with HSVwt and HSVmut, mCMV-specific cytotoxic T cells were indistinguishable between wt and CD11c-Rac mice for mCMVwt as well as mCMVmut (Figure 4E). Therefore we conclude that priming of mCMV-specific T cells crucially depends on CD8α^+^ DCs, but is independent of their cross-priming capacities.

## Discussion

CD8α^+^ DCs are important for immunity as they have the specific ability to produce high levels of IL-12, direct Th1 responses and produce CD8^+^ T cell responses due to their capacity to cross-present exogenous Ag (Shortman and Heath, 2010). In our study we investigated the interdependency of CD8α^+^ DCs, cross- and direct presentation with viral inhibition of immune functions. Such functionally inhibitory immune subversion mechanisms, which affect DCs, have been identified for HSV and mCMV and were deleted in the respective mutant strains selected for the present study. A key factor for HSV with respect to inhibition of DC-functions is *vhs*, which inhibits expression of MHC class I, class II, cytokine and chemokine production (reviewed in Smiley, 2004). Accordingly, *vhs-*null mutations show severely impaired viral pathogenesis and replication in mouse models *in vivo* (Strelow and Leib, 1996; Leib et al., 1999), suggesting that *vhs* is a virulence factor of HSV causing defects of DC-functions and host immunity (reviewed in Smiley, 2004). We find that DCs infected with HSVmut can very well prime CD8^+^ T cells *in vitro*. This finding suggests that also *in vivo* HSVmut-infected cells should be able to prime CD8 T cells directly and do not depend on cross-presentation by non-infected bystander DCs. Our interpretation is corroborated by the finding that in CD11c-Rac mice, which have reduced cross-priming capacities, CD8 T cell responses to HSVmut are normal, and we conclude that they do not depend on cross-priming. As HSVmut-infected Batf3*^−/−^*-mice do not mount HSV-specific CD8 T cell-responses *in vivo*, we assume that the DC-subpopulations absent in these mice are responsible for direct HSV-presentation. In contrast, HSVwt-infected DCs are severely inhibited to prime CD8 T cells *in vitro*, HSVwt-induced CD8 T cell responses are also absent in Batf3*^−/−^*-mice and do depend on cross-presenting DCs, as they are strongly reduced in CD11c-Rac mice. A potential caveat to this explanation could be the fact, that CD11c-Rac mice do not only have a defect in endocytosis and cross-presentation, but also have slightly lower numbers of CD8α^+^ DCs in their spleens (Kerksiek et al., 2005). It is therefore theoretically possible, that the reduced T cell responses rather reflect the reduced numbers of CD8α^+^ DCs than their capacities to cross-present. However, this explanation is unlikely, as responses to HSVmut are identical in both, wt and CD11c-Rac mice, indicating sufficient DC numbers for the induction of optimal CTL-responses.

In contrast, HSVwt-infected DCs can still prime CD8^+^ T cells *in vitro* (Figure 1), albeit with much lowered efficacy. Such “residual” DC-activity could account for the lower T cell response measured in HSVwt-infected CD11c-Rac mice, when cross-presentation is suboptimal. Our results therefore suggest, that (i) CD8α^+^ DCs are responsible for both, direct and cross-priming of HSV-specific CD8^+^ T cells and (ii) that cross-priming becomes more important when the functional inhibition of infected DCs increases. Cross-priming can compensate viral immune evasion very efficiently. As a consequence, CD8 T cell responses to HSVwt and HSVmut become indistinguishable in cross-presentation competent mice.

This observation seems to parallel earlier findings with other herpesviruses such as mCMV, where deletion of genes showed little or no impact on T cell responses in wt mice *in vivo* (Gold et al., 2004; Munks et al., 2007). In the past it has therefore been difficult to establish a role for cross-presentation by using viruses and their specific mutants. For example, removal of mCMV genes affecting MHC class I expression had no effect on the CD8^+^ T cell responses *in vivo* (Munks et al., 2007). The interpretation of these findings were that either directly infected DCs can still prime CD8^+^ T cells efficiently despite viral inhibition, or that cross-priming is so potent that it dominates CD8^+^ T cell priming to mCMV (Snyder et al., 2010), making responses to mutant and wt virus similar (Munks et al., 2007). It has also been discussed that responses induced by mCMVwt and mutant strains in previous studies were similar, because in addition to inhibition of MHC class I presentation, mCMV is known to also inhibit other functions of infected DCs. For example shutting off expression of costimulatory molecules and upregulating inhibitory ligands by mCMV results in failure to prime CD8^+^ T cells *in vitro* (Andrews et al., 2001; Loewendorf et al., 2004; Benedict et al., 2008). However, these inhibitory mechanisms of mCMV have not been entirely identified yet and therefore the responsible viral genes were neither deleted in the respective experiments (Munks et al., 2007) nor in our study. In a recent publication, Torti et al. (2011) analyzed primary and memory CTL-responses to a single deletion mCMV-mutant (mCMVΔ*m157*) in Batf3*^−/−^*-mice. While, similar to our findings, primary CD8 T cell responses to several epitopes were impaired, development of memory T cells rather seemed to depend on direct priming, as they were normal in Batf3*^−/−^*-mice. Although mCMVwt exerts inhibitory effects on DCs *in vitro* in our experiments, these effects did apparently not affect CD8 T cell immunity *in vivo*. Different experimental strategies have suggested that cross-presentation plays a dominant role in CD8 T cell priming during viral infection (Wilson et al., 2006; Snyder et al., 2010), but intravital microscopy could document also direct priming of naive CD8 T cells by DCs infected with vaccinia virus (Hickman et al., 2008), which also strongly inhibits DC-functions (Engelmayer et al., 1999). The fact that the responses in control and CD11c-Rac mice were comparable for both, mCMVwt as well as mCMVmut, suggests that cross-presentation does not contribute substantially to mCMV T cell immunity. Eventually, mCMV-mediated inhibition of DCs is not sufficient to make cross-presentation necessary. In contrast, the lack of responses in Batf3*^−/−^*-mice indicates absolute necessity for (direct) presentation by CD8α^+^ DCs. Accordingly, our findings that Batf3*^−/−^*-mice, which are deficient for CD8α^+^ DCs, cannot mount CD8^+^ T cell responses to neither HSV, HSVmut, mCMV, mCMVmut, underline that this DC-subpopulation is responsible for both – direct priming upon infection and cross-presentation of viral material from infected surrounding cells. However, our findings can eventually not be generalized for all viruses and routes of infection. For example, the “natural” route of infection for HSV is via the skin or mucosa, where other DC-subpopulations will be involved in direct and cross-presentation. In addition, the deleterious effects of viral immune evasion genes may be different in different DC subtypes. Also the interplay of different DC-subpopulation in skin, mucosa and the respective draining lymph nodes may be different from the scenario in the spleen.

The roles direct vs. cross-priming play during viral infection are certainly dependent on the type of virus, its cellular tropism and the route of infection. While our results establish that cross-presentation counteracts effects of viral inhibitory genes on DCs, full understanding of the different contributions of direct vs. cross-presentation will help to improve classical vaccination as well as DC-targeting approaches.

## Materials and Methods

### Animals

CD11c-Rac- (Kerksiek et al., 2005), Batf3*^−/−^*- (Hildner et al., 2008), P14- (Pircher et al., 1989), and gBT-1-mice (Mueller et al., 2002) have been described before and were all maintained on the C57BL/6 background. Mice were infected with virus preparation diluted in 50 μl PBS and sacrificed with CO_2_ at the indicated time points. Mice were bred and housed at the animal facilities of the Institute for Immunology (LMU, Munich, Germany) and treated in accordance with established guidelines of the Regional Ethics Committee of Bavaria. Animal protocols were approved by local authorities.

### Viruses and infections

HSVwt (KOS), HSVmut (ΔUL41,ICP4,22,27), mCMVwt (C3X), mCMVmut (Δm04Δm06Δm152) were prepared and utilized as described previously (Wagner et al., 2002; Lauterbach et al., 2004). Mice were infected intravenously (i.v.) with the amount of virus indicated in the respective Figure legends.

### Cell isolation and purification

Single cell suspensions from spleens or lymph nodes were obtained by mechanical disruption using a pestle followed by enzymatic digestion in serum-free RPMI medium containing Liberase CI (0.42 mg/ml) and DNase I (0.2 mg/ml, both from Roche, Basel, Switzerland) for 20 min at 37°C. Cells were passed through a 70 μm nylon mesh strainer. Cells were counted on a cell counter (Beckman Coulter, Munich, Germany).

### Generation of bone marrow derived dendritic cells

Femurs and tibiae were flushed with IMDM and erythrocytes were lysed by incubation in ACK buffer for 2 min at room temperature. 1 × 10^7^ bone marrow cells were plated in 10 ml IMDM containing 10% heat inactivated FCS, 2 mM glutamine, 100 U/ml penicillin, 100 μg/ml streptomycin sulfate, 50 μM 2-mercaptoethanol, and 20 ng/ml GM-CSF (IMDM complete) in Petri-dishes. On day three suspension cells and loosely adherent cells were dislodged by gentle pipetting and adherent cells were subsequently released by incubation in cold PBS containing 1 mM EDTA. 7.5 × 10^6^ cells were reseeded in 10 ml fresh IMDM complete per 10 cm dish.

### Infection of DC

For *in vitro* studies BMDCs were infected with virus as described previously (Samady et al., 2003) and utilized 48 h post transfection for T cell stimulation assays. DC (5 × 105) from day 7 BMDC-cultures were pelleted at 1,400 rpm for 5 min at room temperature. The DC were then infected at a multiplicity of infection (MOI) of 1, unless otherwise stated, by resuspension in 200 ml of IMDM medium containing 5 × 10^5^ PFU of virus for 1 h at 37°C and 5% CO_2_. One milliliter of IMDM supplemented with granulocyte-macrophage colony-stimulating factor (0.1 mg/ml) was then added, and the DC were incubated at 37°C and 5% CO_2_.

### CFSE labeling

P14 and gB-T1 T cells were purified from lymphocyte cell suspensions by negative selection (CD8 T cell columns; R&D Systems, Minneapolis, MN, USA). For CFSE labeling 1–50 × 10^6^ erythrocyte-free cells from lymph nodes and spleens were washed twice with PBS and labeled with 5 μM CFSE (Molecular Probes, Eugene, OR, USA) for 10 min at 37°C in PBS. After stopping the reaction (PBS, 2% FBS) and washing in PBS, cells were utilized for the *in vitro* assays.

### T cell stimulation

5 × 10^4^ CFSE-labeled P14 and gB-T1 T cells were cultured for 4 days with 2500 DCs, which were either virus infected or loaded with 1 μg/ml of the respective peptide.

### Antibodies and flow cytometry

Lymphocytes were analyzed using anti-CD8a-APC, anti-CD44-FITC, CD19-PerCP, IFNg-PE from Caltag (Burlingame, CA, USA). H-2 K^b^/gB_498–505_-tetramer-PE complexes were purchased from ProImmune Limited. Staining of surface molecules was performed with 1 × 10^6^ to 6 × 10^6^ cells in cold staining buffer for 30 min at 4°C (15 min at room temperature in the dark when MHC multimers were used). Dead-cell exclusion was attained by incubation with 1 μg/ml ethidium monoazide bromide (EMA, Molecular Probes) prior to surface staining or the addition of 0.8 mg/ml propidium iodide (PI, Sigma). Intracellular staining for cytokines was performed with the Cytofix/Cytoperm kit (PharMingen). Flow cytometry was performed with a FACSCalibur or FACSaria (Becton Dickinson), and data were analyzed with FlowJo software (Tree Star).

### Stimulation of cytokine production by epitope-specific T cells

Splenocyte suspensions were prepared according to standard procedures and lymphocytes were stimulated with 1 μg/ml of the indicated peptides in the presence of Brefeldin A (10 μg/ml; Sigma, St. Louis, MO, USA). Cells were surface stained for 30 min at 4°C before the fixation and permeabilization in 500 μl 2x FacsLyse (BD Biosciences) containing 0.05% Tween 20 (Sigma-Aldrich, St. Louis, MO, USA) for 10 min at room temperature. The intracellular staining of cytokines was performed for 30 min at room temperature. Analytic flow cytometry was performed on a FACScanto II (Becton Dickinson). Analysis was performed using FlowJo software (Tree Star, San Carlos, CA, USA).

### Statistical analysis

All statistical analyses were performed using the two-tailed Student’s *t-*test with unequal variance.

## Conflict of Interest Statement

The authors declare that the research was conducted in the absence of any commercial or financial relationships that could be construed as a potential conflict of interest.
